# Immunoglobulin A Vasculitis in a Patient on Hemodialysis and With a Metastatic Liver Lesion

**DOI:** 10.7759/cureus.13863

**Published:** 2021-03-13

**Authors:** Naoki Yamamoto, Akihiro Dejima, Kenkou Hasatani

**Affiliations:** 1 Department of Internal Medicine, Suzu General Hospital, Suzu, JPN

**Keywords:** iga vasculitis, haemodialysis (hd), henoch-schönlein purpura, metastatic liver lesion

## Abstract

We present the case of a 79-year-old man on hemodialysis with immunoglobulin A (IgA) vasculitis. He developed palpable purpura three weeks after having pneumonia. A skin biopsy showed leukocytoclastic vasculitis with IgA and C3 deposition. He received a topical corticosteroid for his IgA vasculitis. He was also diagnosed with a metastatic liver lesion, which was thought to be of colorectal origin because of the elevations in carcinoembryonic antigen and cancer antigen 19-9 levels. The skin biopsy played an important role in the diagnosis of the patient on hemodialysis. Pneumonia and a metastatic liver lesion thought to be from colorectal cancer might be related to the pathogenesis of IgA vasculitis.

## Introduction

Immunoglobulin A (IgA) vasculitis, also known as Henoch-Schönlein purpura (HSP), is a systemic vasculitis characterized by the following tetrad of clinical manifestations: palpable purpura, arthralgia, gastrointestinal symptoms, and renal disease. IgA vasculitis most commonly occurs in children between the ages of four and seven. The incidence rate is estimated to be 14 cases per 100,000 annually [1]. In adults, on the other hand, the annual incidence rate is only 1.3 cases per 100,000, with a mean patient age of 50 years [2]. The American College of Rheumatology diagnostic criteria for HSP include the following: 1) palpable purpura, 2) age of less than 20 years at disease onset, 3) bowel angina, and 4) wall granulocytes on biopsy. HSP can be diagnosed based on the presence of at least two of these criteria with a sensitivity of 87.1% and specificity of 87.7% [3]. In this report, we present a case of IgA vasculitis, which was diagnosed by palpable purpura and a skin biopsy, in a patient on hemodialysis, with recent pneumonia, a metastatic liver lesion, and nephrosclerosis.

## Case presentation

**Figure 1:**
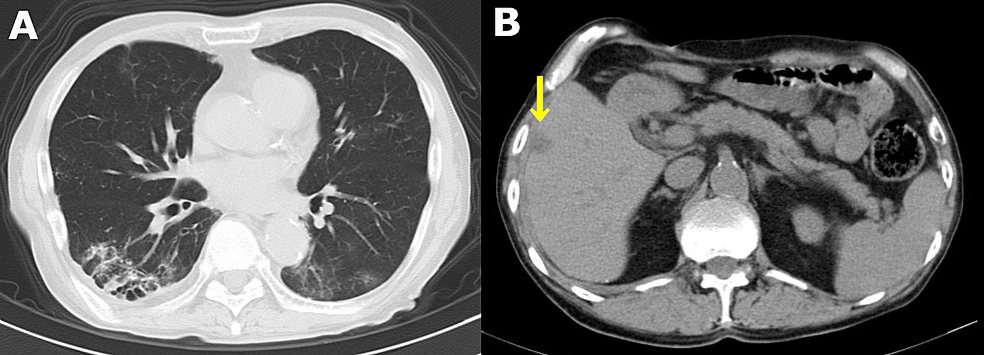
CT findings in the lung and liver A: Mottled gland-glass opacity with an emphysematous change in the bilateral lower lobes. B: SOL in the S5 of the liver (yellow arrow) CT: computed tomography; SOL: space-occupying lesion

A 79-year-old man with a history of colorectal adenocarcinoma and choledocholithiasis was started on hemodialysis a year ago following a CT finding that showed bilateral renal atrophy. He was diagnosed with hypertensive nephrosclerosis at that time; he had been treated with antihypertensives for more than 20 years. He recently presented to the emergency room with epigastralgia, vomiting, and a fever of 39.4 ℃. There was no evidence of a rash, arthralgia, or gross hematuria. Laboratory test results were as follows: white blood cell count: 7.8 x 10^6^/L; neutrophil count: 7.2 x 10^6^/L; hemoglobin level: 11.7 g/dL; platelet count: 16.9 x 10^6^/L; total protein: 7.5 mg/dL; albumin: 3.8 g/dL; aspartate aminotransferase: 34 IU/L; alanine aminotransferase: 35 IU/L; alkaline phosphatase: 370 U/L; gamma-glutamyltransferase: 9 IU/L; and C-reactive protein level: 0.40 mg/dL. CT showed bilateral pneumonia and an enlarged hepatic space-occupying lesion (SOL) (Figure 1).

**Figure 2:**
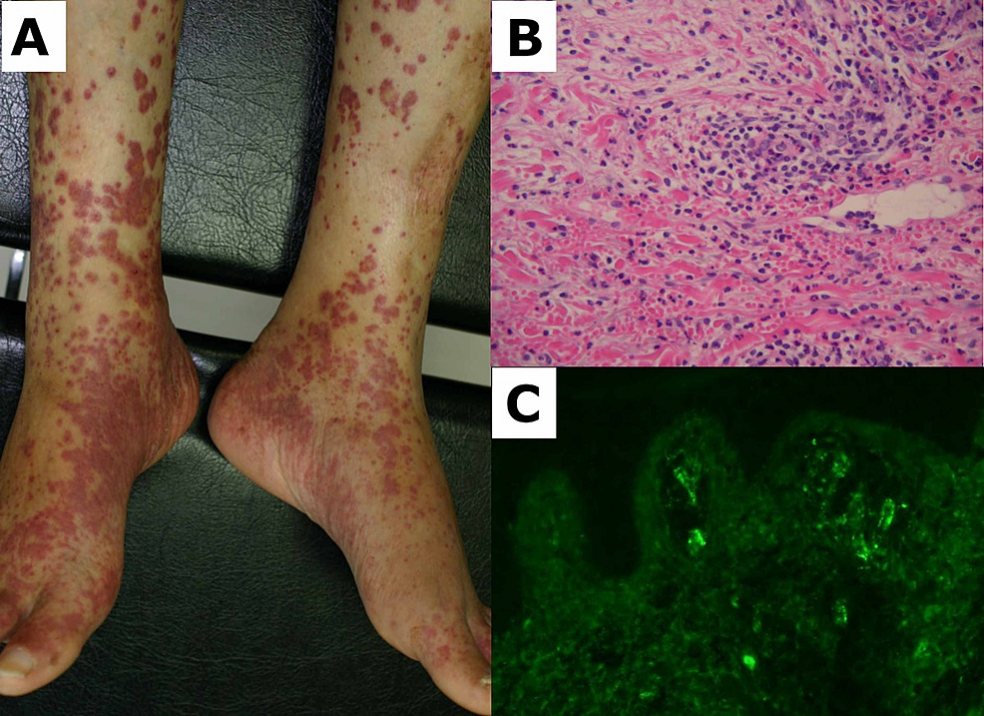
Findings of skin biopsy performed on palpable purpura A: Palpable purpura on the lower limbs. B: Perivascular infiltration of leukocytes and nuclear dust with extravasated erythrocytes (hematoxylin and eosin staining). C: Granular immunoglobulin A deposition within the vascular wall of the dermis (direct immunofluorescence)

He received sulbactam and cefoperazone for pneumonia and cholangitis. After 10 days, he was discharged from the hospital. Three weeks after the onset of his abdominal symptoms, he noticed a rash on his lower limbs. The rash evolved into palpable purpura after a few days (Figure 2A). Additional laboratory studies showed proteinase-3 antineutrophil cytoplasmic antibodies (ANCA) and myeloperoxidase-ANCA almost within the normal limits, at 0.8 IU/mL (normal range: <3.5 IU/mL) and 2.1 IU/mL (normal range: <2.0 IU/mL), respectively. Total protein and albumin were decreased at 6.6 mg/dL and 2.6 g/dL, respectively. Hematoxylin and eosin staining of a skin biopsy showed leukocytoclastic vasculitis (Figure 2B). Immunofluorescence studies showed perivascular deposition of IgA and C3 (Figure 2C).

**Figure 3:**
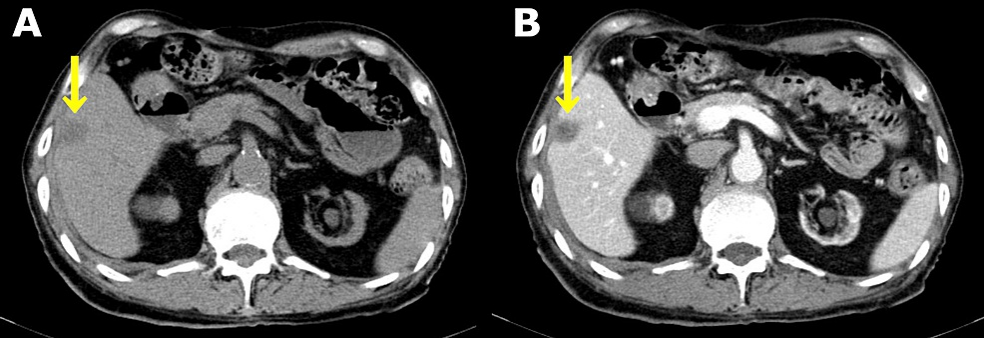
Follow-up CT findings in the liver A: The hepatic SOL in plain CT (yellow arrow). B: The hepatic SOL with a slightly high density in the arterial phase (yellow arrow) CT: computed tomography; SOL: space-occupying lesion

The patient was diagnosed with IgA vasculitis and received topical corticosteroids, after which the palpable purpura gradually disappeared. A follow-up enhanced CT for cancer showed that the hepatic SOL had a slightly high density in the arterial phase (Figure 3).

Laboratory studies showed that alpha-fetoprotein and protein induced by vitamin K absence or antagonist II were negative, but carcinoembryonic antigen and cancer antigen 19-9 were elevated at 38.6 ng/mL and 519.2 U/mL, respectively. The patient was diagnosed with a metastatic liver lesion most likely of an appendiceal or rectal cancer origin; he did not have hepatitis B, hepatitis C, a habit of drinking alcohol, or non-alcoholic steatohepatitis. He declined to undergo surgery and chemotherapy.

## Discussion

IgA vasculitis is an IgA antibody-associated small vessel disease. IgA deposition can be observed in the skin, kidneys, and gastrointestinal tract. In one study of 260 adults with IgA vasculitis, all patients were found to have palpable purpura, 61% had a joint disorder, 70% had renal symptoms, and 53% had gastrointestinal symptoms [4].

Our patient presented with palpable purpura that was proven to be a leukocytoclastic vasculitis with IgA and C3 deposition. A typical gastrointestinal symptom is an abdominal pain. In another study, melena and hematemesis were observed in 18 and two patients, respectively, out of 221 patients of less than 16 years in age with IgA vasculitis [5]. Although gastrointestinal vasculitis is not directly confirmed by endoscopy, it is possible that the patient's epigastralgia and vomiting might have resulted from bowel angina with IgA vasculitis. Palpable purpura typically precedes gastrointestinal symptoms; however, in some cases, it may appear later or not at all [6]. Furthermore, the abdominal symptoms of IgA vasculitis are often overlooked because they are mostly transient and improve without treatment. It may be helpful to measure a serum albumin level to diagnose intestinal involvement in patients without abdominal symptoms [5]. In our case, the patient showed a serum albumin loss from 3.8 to 2.6 g/dL without proteinuria. Renal biopsy was not performed because his bilateral kidneys were atrophic and he was on hemodialysis. There are limited data in the literature/case reports related to IgA vasculitis in patients on hemodialysis [7]. Patients with IgA vasculitis on hemodialysis are less likely to have gross hematuria due to the dysfunction of the glomeruli. Therefore, skin biopsy plays an important role in the diagnosis of these patients.

In the present case, both infection and malignancy could have been triggers for IgA vasculitis. Firstly, the patient had pneumonia before the clinical manifestation of IgA vasculitis. IgA vasculitis is hypothesized to be caused by infections and vaccinations. It is likely that microorganisms have a similar antigenic structure as the blood vessel. Microorganisms could produce cross-reactive antiendothelial cell antibodies, which cause IgA vasculitis. However, specific microorganisms have not yet been detected [8]. Secondly, he had a metastatic lesion of the liver. Paraneoplastic vasculitis accounts for less than 5% of all vasculitis cases [9]. However, 17% of IgA vasculitis patients over 40 years of age had their condition strongly related to malignant tumors [10]. Among IgA vasculitis patients with malignancies, solid tumors accounted for 61% of the malignancies. Patients were mostly over 60 years of age, men, and the most frequent type of solid tumor was lung cancer [11]. Aberrant production of antibodies and tumor antigens, the resemblance of tumor antigens to endothelial cell antigens, and decreased clearance of immune complexes may all potentially play an important role in IgA vasculitis [12]. Screening for malignancies is important in cases of IgA vasculitis in adults, especially in patients on dialysis as they have an increased cancer risk [13].

This was a unique case, and its rarity lies in the fact that a patient with nephrosclerosis on hemodialysis newly developed IgA vasculitis. Infections and malignancies could contribute to the rare combination of IgA vasculitis and hemodialysis. Therefore, we have to investigate whether the patient has underlying infections and malignancies in cases of IgA vasculitis on hemodialysis.

## Conclusions

In this report, we described a case of IgA vasculitis in a patient on hemodialysis and with a metastatic liver lesion who had a history of colorectal adenocarcinoma. When a urine analysis or elevated creatinine cannot be used for the diagnosis due to the patient being on hemodialysis, a skin biopsy can be a critical diagnostic tool in patients suspected of having IgA vasculitis, since the prevalence of gastrointestinal symptoms and arthralgia are lower than that of palpable purpura. In patients who are in their fifth decade of life, it is also essential to screen for underlying infections and malignancies, which could cause IgA vasculitis.
